# Racial/ethnic disparities in de novo metastases sites and survival outcomes for patients with primary breast, colorectal, and prostate cancer

**DOI:** 10.1002/cam4.1322

**Published:** 2018-02-26

**Authors:** Tomi Akinyemiju, Swati Sakhuja, John Waterbor, Maria Pisu, Sean F. Altekruse

**Affiliations:** ^1^ Department of Epidemiology University of Alabama at Birmingham Birmingham Alabama; ^2^ Comprehensive Cancer Center University of Alabama at Birmingham Birmingham Alabama; ^3^ Division of Preventive Medicine University of Alabama at Birmingham Birmingham Alabama; ^4^ Cancer Statistics Branch Division of Cancer Control and Population Sciences National Cancer Institute Bethesda Maryland

**Keywords:** Cancer survival, metastasis, mortality, primary cancer site, racial disparities

## Abstract

Racial disparities in cancer mortality still exist despite improvements in treatment strategies leading to improved survival for many cancer types. In this study, we described race/ethnic differences in patterns of de novo metastasis and evaluated the association between site of de novo metastasis and breast, prostate, and colorectal cancer mortality. Data were obtained from the Surveillance Epidemiology and Ends Results (SEER) database from 2010 to 2013 and included 520,147 patients ages ≥40 years with primary diagnosis of breast, colorectal, or prostate cancer. Site and frequency of de novo metastases to four sites (bone, brain, liver, and lung) were compared by race/ethnicity using descriptive statistics, and survival differences examined using extended Cox regression models in SAS 9.4. Overall, non‐Hispanic (NH) Blacks (11%) were more likely to present with de novo metastasis compared with NH‐Whites (9%) or Hispanics (10%). Among patients with breast cancer, NH‐Blacks were more likely to have metastasis to the bone, (OR: 1.25, 95% CI: 1.15–1.37), brain (OR: 2.26, 95% CI: 1.57–3.25), or liver (OR: 1.62, 95% CI: 1.35–1.93), while Hispanics were less likely to have metastasis to the liver (OR: 0.76, 95% CI: 0.60–0.97) compared with NH‐Whites. Among patients with prostate cancer, NH‐Blacks (1.39, 95% CI: 1.31–1.48) and Hispanics (1.39, 95% CI: 1.29–1.49) were more likely to have metastasis to the bone. Metastasis to any of the four sites evaluated increased overall mortality by threefold (for breast cancer and metastasis to bone) to 17‐fold (for prostate cancer and metastasis to liver). Racial disparities in mortality remained after adjusting for metastasis site in all cancer types evaluated. De novo metastasis is a major contributor to cancer mortality in USA with racial differences in the site, frequency, and associated survival.

## Introduction

Important advancements in the molecular characterization and treatment strategies for the major cancer types have led to significant improvements in survival, with 5‐year relative survival at 99% for prostate cancer, 89% for breast cancer, and 65% for colorectal cancer in 2016 1. However, these improvements mask significant racial disparities in cancer outcomes with experiencing lower survival rates for breast, prostate, and colorectal cancer even after adjusting for demographics, socioeconomic status, access to health care, and prevalence of comorbidities 2, 3, 4, 5, 6, 7. The underlying causes of the persistent racial disparities remain unclear, but likely involve differences in disease aggressiveness, characterized by the frequency, and site of distant metastasis. For most solid tumors, the proximal cause of death is often not the primary tumor, but metastatic spread and systemic disease, accounting for about 90% of cancer‐related deaths 8, 9. Therefore, racial/ethnic differences in the pattern of de novo distant metastasis likely contribute to observed disparities in cancer survival.

For primary tumors, the presence and site of distant metastasis may have important consequences for prognosis, treatment, and subsequent clinical sequelae, influencing survival. For instance, in the absence of distant metastasis in colorectal cancer, surgery with curative intent is a common treatment modality, and adjuvant therapy is rarely essential if there is no evidence of lymphatic involvement 10. Past studies have examined patterns of metastasis and provide evidence that there are differences in the frequency, pattern, and site of distant metastasis for various primary tumors 8, 11, 12, 13, 14. In a comprehensive analysis of 16 major tumor sites, Budczies et al. observed major differences in the frequency of metastasis across sites, with melanoma and breast cancers having the highest mean number of metastasis per case (5.9 and 5.2, respectively), and liver cancer with the lowest mean number of metastasis (2.3) 8. Further, liver, nonregional lymph nodes, lung, and bones were secondary sites most frequently affected (59%, 53%, 44%, and 38%, respectively), while bone marrow, spleen, pancreas, and thyroid were affected less than 5% of the time. Additionally, major differences were observed in the site of distant metastasis by primary tumor site; for instance, breast tumors tended to metastasize to liver, bones, and nonregional lymph nodes (80%, 79%, and 60%, respectively), while prostate cancers tend to metastasize to the bone (91%) 8.

Racial/ethnic differences in the site of de novo metastasis have not been well examined to date. If the pattern of de novo metastasis varies by race/ethnicity, it may point to potential biological mechanisms through which African Americans persistently experience higher mortality rates compared to other racial/ethnic groups. Importantly, by identifying race‐specific patterns of de novo metastasis, especially for highly prevalent cancer types with documented survival disparities, interventions to increase postdiagnosis surveillance and tailored screening strategies may help to prevent distant metastasis, thereby improving the efficacy of primary treatment and increasing survival. In this analysis, we examine the patterns of de novo metastasis for breast, prostate, and colorectal cancer among non‐Hispanic Whites, non‐Hispanic Blacks, and Hispanics represented in the population‐based Surveillance Epidemiology and End Results database.

## Methods

### Data source and study population

Data from the National Cancer Institute Surveillance, Epidemiology and End Results (SEER) database (November 2015 submission) were used for analysis 15. Study participants were non‐Hispanic (NH) White, NH‐Black, and Hispanic adults, aged 40 years and above with a primary diagnosis of breast, colorectal, or prostate cancer represented in the registry and diagnosed between 2010 and 2013. The SEER 18 population‐based dataset includes all incident cancer cases diagnosed in the following SEER cancer registries: Alaska Natives, Atlanta, Georgia; Connecticut; Detroit, Michigan; Hawaii; Iowa; New Mexico; San‐Francisco‐Oakland, California; Seattle, Washington; Utah; Los Angeles, California; San Jose‐Monterey, California; rural Georgia; Greater California, Kentucky, Louisiana, Greater Georgia, and New Jersey. SEER covers about 28% of the U.S. population and includes detailed information for each cancer diagnosis on demographics and clinical characteristics (including stage, grade, lymph node involvements, vital status, and survival months).

### Ethics and consent statement

This study was considered exempt by the Institutional Review Board at the University of Alabama at Birmingham, as the SEER database is a publicly available and non‐identifiable secondary data source.

### De novo metastasis variables

Since 2010, SEER has collected detailed information on the site of distant metastasis among patients with cancer included in the registry. In addition to a metastasis variable representing whether there is any evidence of metastasis, the site of metastasis to four major sites is recorded, including lung, liver, brain, and bone. Metastasis to any of those sites may also include metastasis to any of the other three sites or to other sites not indicated. For the current analysis, we evaluated racial differences in the presence of de novo metastasis (i.e., distant metastasis at presentation) and the association between de novo metastasis to specific sites and overall survival. A total of 207,167 breast, 123,795 colorectal, and 189,185 prostate cancer patients were included in this analysis. Patients with metastasis site unknown and those with no data on metastasis site were excluded from analysis, that is, 1685 patients with breast cancer, 5452 patients with colorectal cancer, and 1063 patients with prostate cancer.

### Outcome variables

The primary outcomes of interest for this study were racial disparities in (1) site of de novo metastasis and (2) overall survival associated with de novo metastasis. We assessed the risk of cancer mortality associated with site of de novo metastasis (lung, liver, bone, brain, or any) for each primary cancer site.

### Statistical analysis

We described baseline socio‐demographic, clinical, and treatment variables among study participants by race/ethnicity using chi‐square tests. In addition, we conducted a descriptive analysis of the frequency and site of de novo metastasis for each primary site stratified by race/ethnicity to examine the proportion of any de novo metastasis, as well as de novo metastasis to each of lung, liver, brain, and bone sites by race/ethnicity. We used logistic regression analysis to examine racial differences in de novo metastasis, adjusted for age, marital status, and sex. As stage of diagnosis was highly correlated with distant metastasis, this variable was not included in multivariable models. We fit extended Cox regression models adjusted for age, marital status, treatment, and stage to estimate the adjusted hazard ratios associated with site of de novo metastasis by race. We modeled time to cancer‐related death as the outcome and censored patients at the time of death, or end of follow‐up (December 2013). For outcomes related to colorectal cancer, we adjusted for sex in addition to age, marital status, treatment, and stage. We adjusted for employment, poverty, and education in additional analysis; however, given that these variables were highly missing in the SEER dataset and results were consistent, the less adjusted models are presented in the results. Kaplan–Meier curves were generated for each metastatic site to estimate probability of survival stratified by race/ethnicity, and tests of proportionality were conducted by including interaction terms for race*survival time in the models.

## Results

A total of 520,147 breast, colorectal, and prostate cancer patients aged 40 years and older were identified in SEER database between 2010 and 2013 (Table 1). A higher proportion of NH‐Blacks and Hispanics presented with de novo metastasis compared with NH‐Whites for all cancer types considered. Hispanics with breast or prostate cancer were slightly less likely to receive radiation therapy compared with NH‐Whites or NH‐Blacks (breast: 46% vs. 50% and 49%, prostate: 32% vs. 33% and 39%); however, receipt of surgery was significantly lower among NH‐Blacks compared to NH‐Whites and Hispanics for all three cancers (breast: 86% vs. 92% and 90%, colorectal: 80% vs. 84% and 82%, and prostate 34% vs. 43% and 41%) . There were significant racial differences in de novo metastasis, observed among 11% of NH‐Blacks, 10% of Hispanics, and 9% of NH‐Whites (*P *< 0.001).

**Table 1:** Patient socio‐demographic and clinical characteristics by race/ethnicity, SEER 2010–2013

	Primary Cancer type								
	Breast cancer^a^ (*N *= 207,167)			Colorectal cancer^b^ (*N *= 123,795)			Prostate cancer^c^ (*N *= 189,195)		
	NH‐Blacks 11.61%	NH‐Whites 77.20%	Hispanics 11.19%	NH‐Blacks 13.39%	NH‐Whites 75.17%	Hispanics 11.45%	NH‐Blacks 16.34%	NH‐Whites 73.88%	Hispanics 9.78%
Age (in years)									
40–49	19.41	13.88	25.65	10.28	7.16	12.55	5.51	2.31	3.59
50–59	28.94	23.47	29.41	26.43	18.48	25.83	28.52	19.91	22.68
60–69	27.09	28.74	24.32	28.60	24.35	25.61	41.61	42.70	40.36
70–79	16.28	20.56	13.92	21.34	24.69	20.51	20.01	26.46	25.81
80+	8.29	13.35	6.69	13.34	25.33	15.51	4.35	8.62	7.55
Sex									
Female	–	–	–	49.83	48.00	46.46	–	–	–
Male	–	–	–	50.17	52.00	53.54	–	–	–
Married									
Never married	27.78	11.13	17.61	27.41	12.16	27.41	19.39	8.36	10.62
Ever married	72.22	88.87	82.39	72.59	87.84	72.59	80.61	91.64	89.38
De novo metastasis									
No	91.38	94.55	91.38	75.34	80.28	78.81	93.68	94.8	93.15
Yes	8.62	5.45	8.62	24.66	19.72	21.19	6.32	5.20	6.85
Surgery									
Yes	86.31	91.70	90.23	80.00	83.97	81.82	33.92	43.43	40.83
No	13.58	8.18	9.70	19.72	15.79	18.05	65.58	56.05	58.80
Unknown	0.11	0.12	0.07	0.28	0.24	0.24	0.50	0.53	0.37
Radiation									
Yes	48.92	49.97	46.49	10.81	13.39	14.92	38.80	32.72	32.32
No	50.24	49.36	53.12	88.66	85.90	84.79	60.34	66.33	67.20
Unknown	0.84	0.67	0.38	0.53	0.71	0.28	0.86	0.95	0.48
Died									
No	88.02	92.32	93.67	69.64	72.23	75.79	92.58	93.45	93.89
Yes	11.98	7.68	6.33	30.36	27.77	24.21	7.42	6.55	6.11

All *P* < 0.0001.

a Female patients with breast cancer.

b Male and female patients with colorectal cancer.

c Male patients with prostate cancer.

The pattern of de novo metastasis within each primary site by race/ethnicity is presented in Table 2. Among patients with breast cancer, 9% of NH‐Blacks, 6% of NH‐Whites, and 6% of Hispanics presented with de novo metastasis. Among patients with breast cancer, the most common de novo metastasis site was to the bone (3%, 2%, and 2% for NH‐Blacks, NH‐Whites, and Hispanics, respectively); for colorectal cancer, the most common site was to the liver (15%, 11%, and 12% for NH‐Blacks, NH‐Whites, and Hispanics, respectively); and for prostate, the most common site was also to the bone (5%, 4%, and 5% for NH‐Blacks, NH‐Whites, and Hispanics, respectively). About a third of all patients with primary breast cancer had at least two de novo metastases, compared with about 20% for patients with colorectal cancer and 10% for patients with prostate cancer.

**Table 2:** Pattern of de novo metastasis by race/ethnicity and primary site, SEER 2010‐2013

Primary site ‐ metastatic site	Race/ethnicity [*N* (%)^a^]		
Any metastasis	NH‐Blacks 11.3%	NH‐Whites 8.7%	Hispanics 9.7%
Breast (*N* = 207,167)			
No metastasis	21982 (91.38)	151216 (94.55)	21892 (94.33)
Any metastasis (*N* = 12,077)^c^	2073 (8.62)	8712 (5.45)	1292 (5.57)
To bone	647 (2.89)	3372 (2.18)	459 (2.05)
To brain	42 (0.19)	115 (0.08)	23 (0.10)
To liver	160 (0.72)	633 (0.42)	73 (0.33)
To lung	261 (1.17)	829 (0.55)	145 (0.66)
Number of metastatic sites^d^			
0^b^	313 (15.10)	1166 (13.38)	206 (15.94)
1	1110 (53.55)	4949 (56.81)	700 (54.18)
2–4	650 (31.36)	2597 (29.81)	386 (29.88)
Colorectal (*N* = 123,795)			
No metastasis	12485 (75.34)	74703 (80.28)	11169 (78.81)
Any metastasis^c^ (*N* = 25,438)	4086 (24.66)	18349 (19.72)	3003 (21.19)
To bone	41 (0.33)	212 (0.28)	43 (0.38)
To brain	14 (0.11)	77 (0.10)	7 (0.06)
To liver	2168 (14.80)	9489 (11.27)	1543 (12.14)
To lung	231 (1.82)	1100 (1.45)	186 (1.64)
Number of metastatic sites^d^			
0^b^	735 (17.99)	4048 (22.06)	669 (22.28)
1	2454 (60.06)	10878(59.28)	1779 (59.24)
2–4	897 (21.95)	3423 (18.65)	555 (18.48)
Prostate (*N* = 189,195)			
No metastasis	28956 (93.68)	132508 (94.80)	17238 (93.15)
Any metastasis^c^ (*N* = 10,483)	1952 (6.32)	7264 (5.20)	1267 (6.85)
To bone	1533 (5.03)	5658 (4.10)	982 (5.39)
To brain	3 (0.01)	11 (0.02)	3 (0.01)
To liver	21 (0.07)	63 (0.05)	10 (0.06)
To lung	26 (0.09)	18 (0.07)	97 (0.10)
Number of metastatic sites^d^			
0^b^	162 (8.30)	777 (10.70)	124 (9.79)
1	1538 (81.10)	5829 (80.25)	1013 (79.95)
2–4	207 (10.60)	658 (9.06)	130 (10.26)

a Column percent.

b 0 number of metastatic sites reflects unknown/not applicable.

c Among those with de novo metastasis documented.

d Among those with any de novo metastasis, number of metastasis sites documented (from bone, brain, liver, or lung), else 0, which may include metastasis to other distant sites.

Racial differences in site of de novo metastases were observed after adjusting for age, marital status, and sex (for colorectal cancer) (Table 3). NH‐Black patients with primary breast cancer were more likely to have de novo metastasis to the bone (OR: 1.25, 95% CI: 1.15–1.37), brain (OR: 2.26, 95% CI: 1.57–3.25), or liver (OR: 1.62, 95% CI: 1.35–1.93) compared with NH‐Whites. However, among patients with primary prostate cancer, NH‐Blacks and Hispanics each had 39% higher odds for de novo metastasis to bone (NH‐Blacks: OR: 1.39, 95% CI: 1.31–1.48, Hispanics: OR: 1.39, 95% CI: 1.29–1.49), while NH‐Blacks also had 83% higher odds for de novo metastasis to liver (OR: 1.83, 95% CI: 1.10–3.03) compared to NH‐Whites. NH‐Black colorectal cancer patients were at 30% higher odds for developing de novo metastasis to the liver (OR: 1.30, 95% CI: 1.23–1.37) and 24% higher odds for de novo metastasis to lungs (OR: 1.24, 95% CI: 1.07–1.43) compared to NH‐Whites. NH‐Blacks with primary breast, colorectal, or prostate cancer were more likely to have de novo metastasis to any site compared with NH‐Whites.

**Table 3:** Multivariable regression analysis for the association between race/ethnicity and site of de novo metastasis, SEER 2010‐2013

Primary cancer site	Race/ethnicity	Metastatic site				
		Bone OR (95% CI)^a^	Brain OR (95% CI)^a^	Liver OR (95% CI)^a^	Lung OR (95% CI)^a^	Any site OR (95% CI)^a^
Breast	NH‐Whites	Ref	Ref	Ref	Ref	Ref
	NH‐Blacks	**1.25 (1.15**–**1.37)**	**2.26 (1.57**–**3.25)**	**1.62 (1.35**–**1.93)**	**2.26 (1.96**–**2.61)**	**1.55 (1.48**–**1.64)**
	Hispanics	0.95 (0.86–1.05)	1.33 (0.85–2.09)	**0.76 (0.60**–**0.97)**	**1.39 (1.16** –**1.66)**	1.04 (0.98–1.10)
Colorectal b	NH‐Whites	Ref	Ref	Ref	Ref	Ref
	NH‐Blacks	1.16 (0.82–1.63)	1.12 (0.63–1.99)	**1.30 (1.23**–**1.37)**	**1.24 (1.07**–**1.43)**	**1.26 (1.21**–**1.31)**
	Hispanics	1.34 (0.96–1.86)	0.63 (0.29–1.37)	1.04 (0.98–1.10)	1.14 (0.98–1.34)	1.04 (1.00–1.09)
	NH‐Whites	Ref	Ref	Ref	Ref	Ref
Prostate	NH‐Blacks	**1.39 (1.31**–**1.48)**	1.67 (0.46–6.06)	**1.83 (1.10**–**3.03)**	1.42 (0.91–2.20)	**1.37 (1.30**–**1.45)**
	Hispanics	**1.39 (1.29**–**1.49)**	2.19 (0.61–7.86)	1.27 (0.65–2.48)	1.48 (0.89–2.45)	**1.39 (1.31**–**1.48)**

Bold indicates level of statistical significance achieved at α =0.05.

Each model assessing odds of metastasis to each of the four sites (bone, brain, liver, or lung) versus no metastasis in relation to race/ethnicity.

a Adjusted for age, marital status.

b Adjusted for age, marital status, sex.

**Figure 1:**
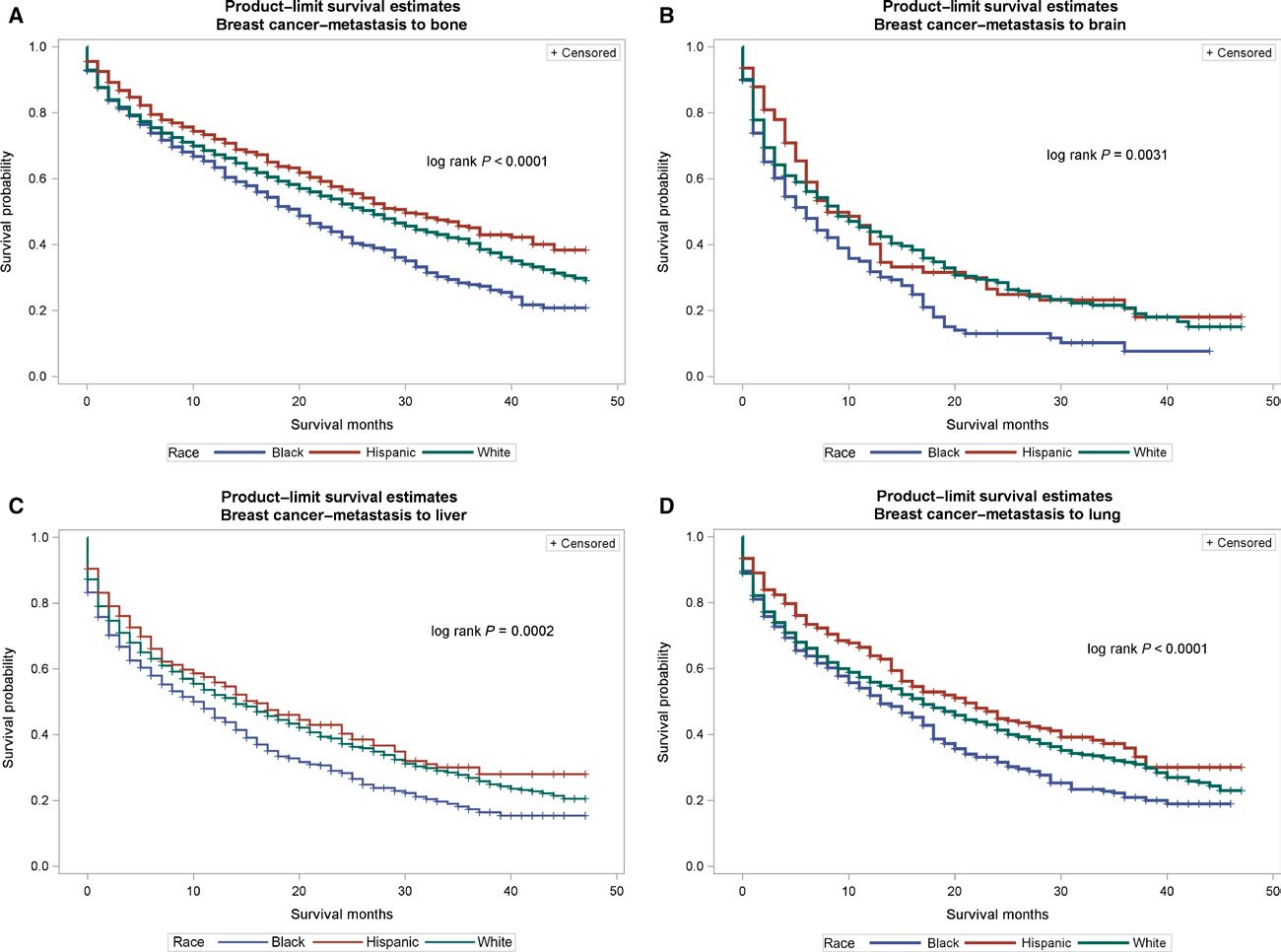
(A–D) Survival curves for de novo metastasis from the primary site to metastatic sites by race/ethnicity for breast cancer. (A) Breast cancer to bone. (B) Breast cancer to brain. (C) Breast cancer to liver. (D) Breast cancer to lung.

**Figure 2:**
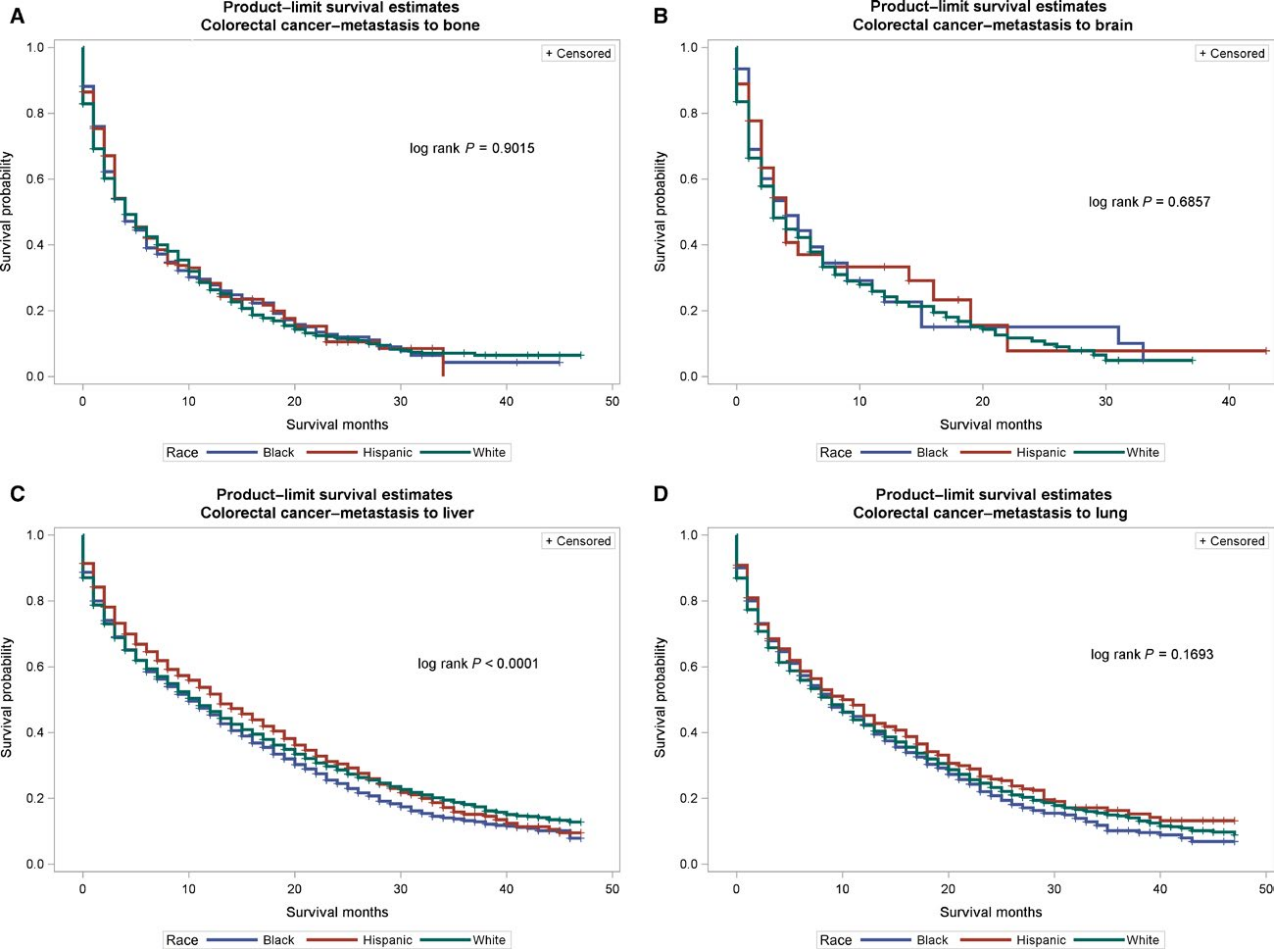
(A–D) Survival curves for de novo metastasis from the primary site to metastatic sites by race/ethnicity for colorectal cancer. (A) Colorectal cancer to bone. (B) Colorectal cancer to brain. (C) Colorectal cancer to liver. (D) Colorectal cancer to lung.

**Figure 3:**
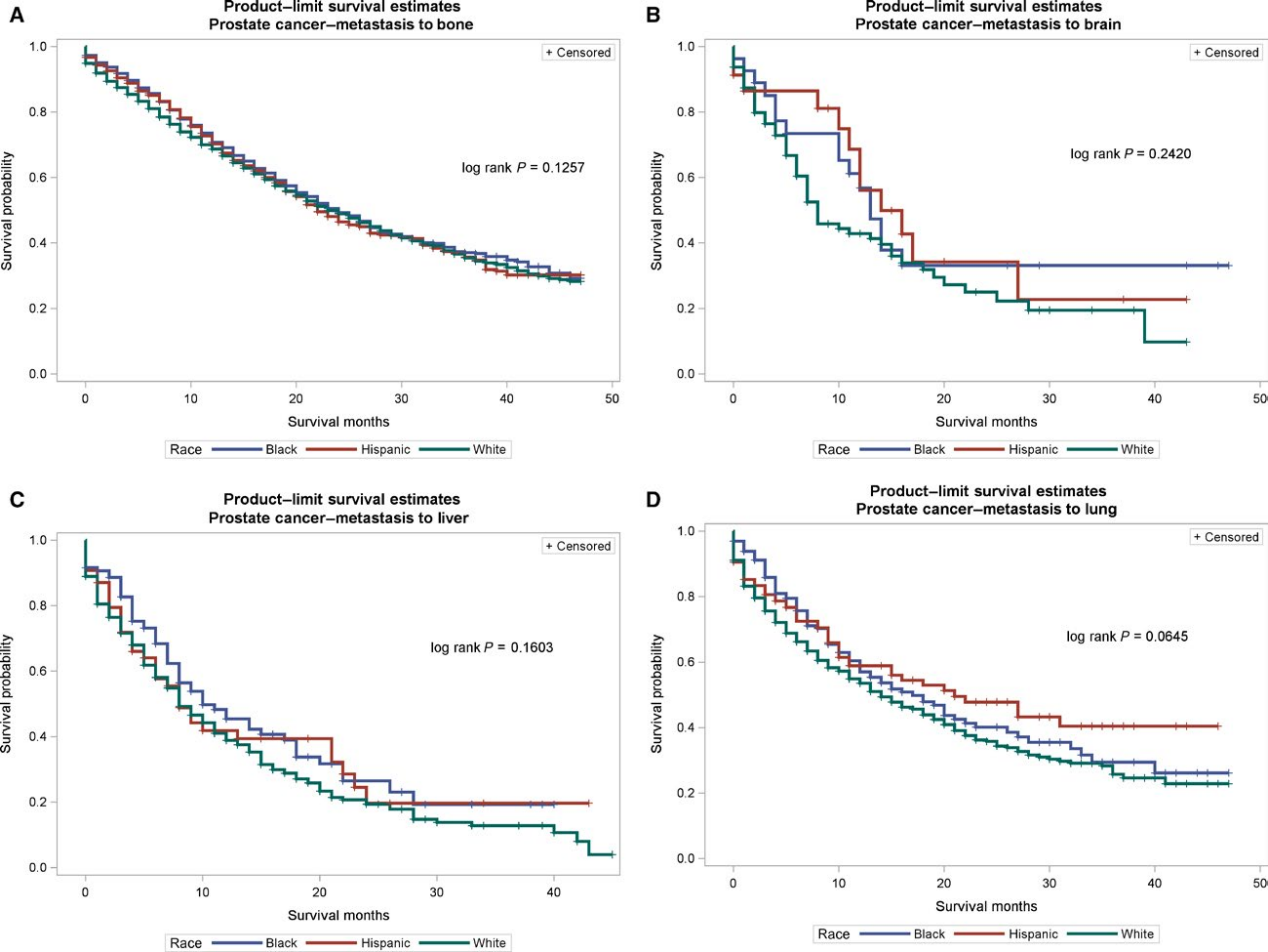
(A–D) Survival curves for de novo metastasis from the primary site to metastatic sites by race/ethnicity for prostate cancer. (A) Prostate cancer to bone. (B) Prostate cancer to brain. (C) Prostate cancer to liver. (D) Prostate cancer to lung.

The probability of survival (Figures 1, 2, 3) among primary cancer patients with de novo metastasis varied by race/ethnicity. For instance, NH‐Blacks with primary breast cancer and de novo metastasis to the bone, brain, liver, and lung had significantly lower survival compared with NH‐Whites and Hispanics (log rank p value for bone: <0.0001, brain = 0.0031, liver = 0.0002, lung < 0.0001). In the Cox regression models (Table 4), de novo metastasis to any of the four de novo metastasis sites was associated with threefold to 17‐fold higher hazards of mortality compared with no metastasis, although the magnitude of the associations varied by primary tumor site and race/ethnicity. For instance, among patients with primary breast cancer, NH‐Blacks had 50% higher hazards for mortality compared to NH‐Whites (HR: 1.50, 95% CI: 1.43–1.58), and de novo metastasis to the bone increased the risk for mortality by threefold (HR: 3.15, 95% CI: 2.97–3.35). De novo metastasis to the brain was also associated with over a 10‐fold increased risk for mortality (HR: 10.17, 95% CI: 8.46–12.23), metastasis to the liver was associated with a sixfold increased risk for mortality (HR: 6.17, 95% CI: 5.57–6.83), and metastasis to the lung was associated with almost fourfold increased hazard for mortality (3.97, 95% CI: 3.54–4.33). NH‐Blacks remained at higher risk of cancer mortality in these models compared with NH‐Whites after accounting for de novo metastasis site. Similar patterns were observed for other primary cancer sites and strikingly, among patients with prostate cancer, de novo metastasis to the liver was associated with a 17‐fold increased hazard of cancer mortality (HR: 17.12, 95% CI: 13.29–22.06). Among primary patients with breast cancer, there were no significant differences between Hispanics and NH‐Whites; however, Hispanics with primary colorectal and prostate cancer had significantly lower hazard for cancer mortality after accounting for de novo metastasis.

**Table 4:** Multivariable adjusted hazards ratios (HR) for the association between race/ethnicity and site of de novo metastasis with survival, SEER 2010‐2013

Main Exposure	Primary cancer site					
	Breast^a^ HR (95% CI)		Colorectal^b^ HR (95% CI)		Prostate^a^ HR (95% CI)	
	Model 1^c^	Model 2^d^	Model 1^c^	Model 2^d^	Model c	Model 2^d^
NH‐Whites	Ref	Ref	Ref	Ref	Ref	Ref
NH‐Blacks	**1.24 (1.09**–**1.42)**	**1.50 (1.43**–**1.58)**	0.82 (0.54–1.25)	**1.15 (1.10**–**1.20)**	1.07 (0.98–1.17)	**1.32 (1.25**–**1.38)**
Hispanics	0.97 (0.81–1.15)	0.99 (0.92–1.05)	1.37 (0.94–2.00)	**0.92 (0.88**–**0.97)**	1.06 (0.95–1.18)	**0.96 (0.90**–**1.03)**
Bone metastasis	–	**3.15 (2.97**–**3.35)**	–	**4.15 (3.64**–**4.75)**	–	**8.18 (7.82**–**8.56)**
NH‐Whites	Ref	Ref	Ref	Ref	Ref	Ref
NH‐Blacks	1.55 (0.97–2.46**)**	**1.56 (1.48**–**1.64)**	1.42 (0.74–2.75)	**1.15 (1.10**–**1.21)**	–	**1.44 (1.36**–**1.53)**
Hispanics	1.67 (0.95–2.92)	0.98 (0.92–1.05)	**2.77 (1.20**–**6.41)**	**0.92 (0.87**–**0.97)**	–	**0.89 (0.82**–**0.97)**
Brain metastasis	–	**10.17 (8.46**–**12.23)**	–	**5.24 (4.20**–**6.54)**	–	**8.69 (3.90**–**19.34)**
NH‐Whites	Ref	Ref	Ref	Ref	Ref	Ref
NH‐Blacks	**1.56 (1.48**–**1.64)**	**1.58 (1.50**–**1.66)**	**1.14 (1.07**–**1.21)**	**1.15 (1.11**–**1.19)**	0.91 (0.47–1.78)	**1.43 (1.35**–**1.52)**
Hispanics	0.97 (0.90–1.03)	1.00 (0.94–1.06)	0.98 (0.91–1.05)	**0.93 (0.90**–**0.97)**	1.67 (0.74–3.80)	**0.89 (0.82**–**0.97)**
Liver metastasis	–	**6.17 (5.57**–**6.83)**	–	**3.80 (3.68**–**3.92)**	–	**17.12 (13.29**–**22.06)**
NH‐Whites	Ref	Ref	Ref	Ref	Ref	Ref
NH‐Blacks	**1.53 (1.25**–**1.87)**	**1.57 (1.49**–**1.65)**	**0.80 (0.65**–**0.99)**	**1.14 (1.09**–**1.19)**	1.42 (0.62–3.25)	**1.45 (1.36**–**1.53)**
Hispanics	1.09 (0.83–1.43)	0.99 (0.93–1.06)	0.83 (0.66–1.05)	**0.91 (0.87**–**0.96)**	0.37 (0.11–1.23)	**0.88 (0.81**–**0.96)**
Lung metastasis	–	**3.97 (3.64**–**4.33)**	–	**2.07 (1.93**–**2.23)**	–	**6.01 (4.56**–**7.92)**
NH‐Whites	Ref	Ref	Ref	Ref	Ref	Ref
NH‐Blacks	**1.37 (1.28**–**1.46)**	**1.41 (1.35**–**1.47)**	**1.11 (1.07**–**1.16)**	**1.09 (1.05**–**1.12)**	1.03 (0.95–1.12)	**1.18 (1.13**–**1.24)**
Hispanics	0.97 (0.89–1.07)	0.96 (0.91–1.01)	0.97 (0.92–1.02)	**0.92 (0.89**–**0.96)**	1.00 (0.91–1.10)	**0.92 (0.86**–**0.98)**
Any metastasis	–	**4.71 (4.51**–**4.92)**	–	**4.03 (3.93**–**4.13)**	–	**9.00 (8.64**–**9.39)**

Bold indicates level of statistical significance achieved at α =0.05.

a Adjusted for age, marital status, treatment and stage.

b Adjusted for age, marital status, sex, treatment and stage.

c Calculated among those with primary cancer type and de novo metastasis to the specified site to evaluate race/ethnic differences in survival.

d Calculated among those with cancer to primary site regardless of metastasis site to evaluate influence of race/ethnic and de novo metastasis on survival.

Missing HR for prostate cancer due to low cell count.

## Discussion

In the large population‐based SEER cancer registry, the site and number of de novo metastasis appeared to vary by race/ethnicity among patients with a primary diagnosis of breast, colorectal, and prostate cancer, with 11% NH‐Blacks, 9% NH‐Whites, and 10% Hispanics diagnosed with de novo metastasis to at least one distant site in the body. De novo metastasis was most prevalent among patients with primary colorectal cancer (20–25%) and least common among patients with prostate cancer (5–7%). The most common de novo metastasis site for primary breast cancer was bone and lung; for primary colorectal cancer, it was liver and lung; and for prostate cancer, it was bone. The hazard of mortality among patients with de novo metastasis to bone, brain, liver, or lung was significantly higher compared with patients with no metastasis, with estimates ranging from a threefold increase to 17‐fold increase in risk of mortality. The relative contribution of de novo metastasis to mortality also appeared to vary by race/ethnicity and primary tumor type. NH‐Blacks had significantly higher risk of mortality compared with NH‐Whites regardless of primary cancer type, even after accounting for site of de novo metastasis and other clinical variables.

Other studies have described patterns of recurring metastasis and the impact of metastasis site on cancer outcomes. For instance, breast cancer was observed to preferentially metastasize to bone and lungs and less commonly to brain and liver 16, but site may vary by breast cancer subtypes. Mitry et al. demonstrated that metastasis to liver was associated with better survival compared to metastasis to any other distant metastatic sites including lungs for patients with primary diagnosis of colorectal cancer 17, and a recent study by Khattak et al. showed better prognosis in colorectal cancer patients with distant metastasis to lungs only when comparing single organ metastasis 18. Prostate cancer has been shown to have a strong predilection for distant metastasis to bone 19, 20 and cancer mortality often reported within 3–5 years once diagnosis of metastasis is established 21. Although none of these prior studies specifically evaluated racial differences, these findings taken together suggest that renewed focus on postdiagnostic surveillance, regardless of primary tumor site, could help with clinical strategies and provide insight relevant to postdiagnosis surveillance and treatment decisions, such as tailoring the frequency and site of screening for recurrent cancer, and proactive anticipation of potential metastasis sites.

Evidence suggests that over 90% of cancer deaths occur due to tumor invasion and metastasis 8, 9. The higher prevalence of late‐stage diagnosis among NH‐Blacks relative to other racial groups 22, 23, our observation that NH‐Blacks were more likely to have de novo metastasis to multiple sites, as well as the strong, independent effects of both race/ethnicity, and de novo metastasis on cancer survival, suggests that other factors remain important in the well‐documented disparities in cancer survival. Multiple studies have suggested that upstream factors such as access to health care, routine screening, and socioeconomic status contribute to the late stage at diagnosis and higher cancer mortality experienced among NH‐Black cancer patients 2, 24, 25, 26. In this study, there were racial differences in stage at diagnosis which may be partially due to racial differences in access to routine health care, associated with routine cancer screening, and early detection of cancer. Lack of screening data in the SEER dataset precluded direct examination of these factors in the current analysis. There are likely also molecular, biochemical, cellular, and/or genetic factors that may predispose NH‐Blacks to faster growing, metastasizing tumors 27, 28. Such biological factors may include altered metabolic and/or inflammatory profiles associated with underlying comorbid conditions such as obesity, diabetes, and chronic stress, conditions more common among NH‐Blacks, and more likely to be associated with adverse cancer outcomes 29, 30, 31, 32. Ongoing work in this area suggests that the invasion‐metastasis cascade involves multiple steps, beginning with local invasion, movement of cancer cells into nearby blood and lymphatic vessels, transit of cancer cells through the lymphatic and circulatory system, escape of cancer cells from vessels into distant tissues, formation of small nodules of cancer cells, and finally the growth of micrometastatic lesions into macroscopic tumors, also known as “colonization” 33. Multiple transcriptional factors are implicated in the tumor migratory process, and inflammatory cells are implicated in cancer cell invasion and growth 34, 35. While there are major unanswered questions remaining regarding the exact mechanisms underlying tumor invasion and metastasis, epidemiologic studies such as this may help to provide important information about the differential patterns and impact of distant metastasis on cancer outcomes, and provide input into clinical decisions regarding surveillance and treatment.

The strength of this study is the use of the population‐based SEER registry to assess site‐specific cancer outcomes and site of de novo metastasis. Additionally, to our knowledge, this is the first attempt to use the SEER dataset and information on de novo metastatic sites to examine racial disparities in cancer outcomes. There are a few limitations to our study as well. The SEER cancer registry does not routinely include data on comorbidities and recurrence, which may influence choice of treatment and cancer survival. Studies have shown that recurrence of distant metastasis can occur after several years of treatment, and may influence survival outcomes 36. Furthermore, detailed data on de novo metastasis were only available on the four sites evaluated here, although these accounted for a significant proportion of metastasis, we were unable to evaluate metastasis to other sites in relation to survival. There was limited data on specific treatment modalities beyond the receipt of surgery and/or chemotherapy, and this may have limited our ability to adjust for treatment differences, which are known to be important for racial disparities in cancer survival. In addition, lack of data on screening frequency and/or modality in the SEER dataset inhibited our ability to examine the proportion of racial differences observed in de novo metastasis that were due to differences in early detection or biological factors.

In conclusion, de novo metastasis is a significant risk factor for cancer mortality among patients with a primary diagnosis of breast, colorectal, and prostate cancer, with racial differences in the site and frequency of metastasis; racial disparities in cancer survival remain after accounting for site of de novo metastasis.

## Conflict of Interest

The authors declare that they have no conflict of interest.
